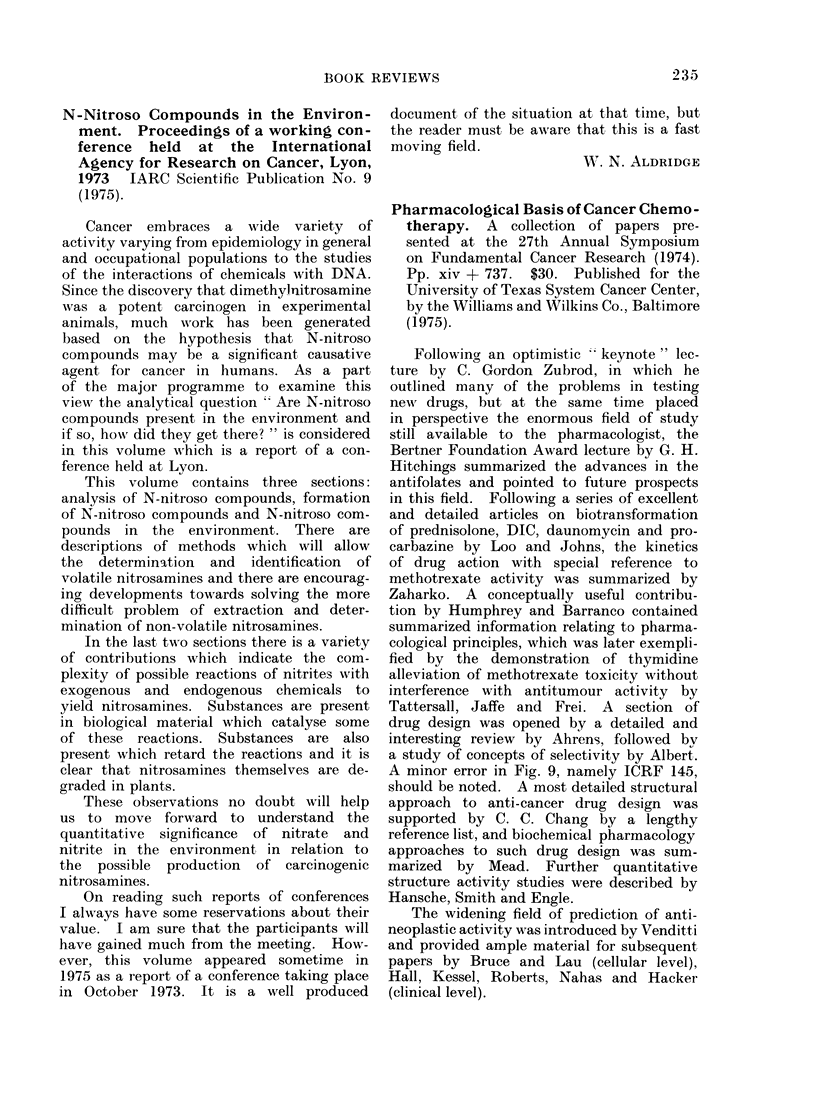# N-Nitroso Compounds in the Environment. Proceedings of a working conference held at the International Agency for Research on Cancer, Lyon, 1973

**Published:** 1976-02

**Authors:** W. N. Aldridge


**N-Nitroso Compounds in the Environment. Proceedings of a working conference held at the International Agency for Research on Cancer, Lyon, 1973** IARC Scientific Publication No. 9 (1975).

Cancer embraces a wide variety of activity varying from epidemiology in general and occupational populations to the studies of the interactions of chemicals with DNA. Since the discovery that dimethylnitrosamine was a potent carcinogen in experimental animals, much work has been generated based on the hypothesis that N-nitroso compounds may be a significant causative agent for cancer in humans. As a part of the major programme to examine this view the analytical question " Are N-nitroso compounds present in the environment and if so, how did they get there? " is considered in this volume which is a report of a conference held at Lyon.

This volume contains three sections: analysis of N-nitroso compounds, formation of N-nitroso compounds and N-nitroso compounds in the environment. There are descriptions of methods which will allow the determination and identification of volatile nitrosamines and there are encouraging developments towards solving the more difficult problem of extraction and determination of non-volatile nitrosamines.

In the last two sections there is a variety of contributions which indicate the complexity of possible reactions of nitrites with exogenous and endogenous chemicals to yield nitrosamines. Substances are present in biological material which catalyse some of these reactions. Substances are also present which retard the reactions and it is clear that nitrosamines themselves are degraded in plants.

These observations no doubt will help us to move forward to understand the quantitative significance of nitrate and nitrite in the environment in relation to the possible production of carcinogenic nitrosamines.

On reading such reports of conferences I always have some reservations about their value. I am sure that the participants will have gained much from the meeting. However, this volume appeared sometime in 1975 as a report of a conference taking place in October 1973. It is a well produced document of the situation at that time, but the reader must be aware that this is a fast moving field.

W. N. Aldridge